# Disease Affects *Bdnf* Expression in Synaptic and Extrasynaptic Regions of Skeletal Muscle of Three SBMA Mouse Models

**DOI:** 10.3390/ijms20061314

**Published:** 2019-03-15

**Authors:** Katherine Halievski, Samir R. Nath, Masahisa Katsuno, Hiroaki Adachi, Gen Sobue, S. Marc Breedlove, Andrew P. Lieberman, Cynthia L. Jordan

**Affiliations:** 1 Neuroscience Program, 108 Giltner Hall, Michigan State University, East Lansing, MI 48824-1115, USA; breedsm@msu.edu (S.M.B.); jordancy@msu.edu (C.L.J.); 2 Department of Pathology, University of Michigan Medical School, Ann Arbor, MI 48109, USA; nathsr@med.umich.edu (S.R.N.); liebermn@med.umich.edu (A.P.L.); 3 Department of Neurology, Nagoya University Graduate School of Medicine, 65 Tsurumai-cho, Showa-ku, Nagoya 466-8550, Japan; ka2no@med.nagoya-u.ac.jp (M.K.); sobueg@med.nagoya-u.ac.jp (G.S.); 4 Department of Neurology, University of Occupational and Environment Health School of Medicine, 1-1 Iseigaoka, Yahatanishi-ku, Kitakyushu Fukuoka 807-8555, Japan; hiadachi@med.uoeh-u.ac.jp; 5 Physiology Department, 108 Giltner Hall, Michigan State University, East Lansing, MI 48824-1115, USA

**Keywords:** SBMA, synaptic, extrasynaptic, gene expression, neurotrophic factors, muscle

## Abstract

Spinal bulbar muscular atrophy (SBMA) is a slowly progressive, androgen-dependent neuromuscular disease in men that is characterized by both muscle and synaptic dysfunction. Because gene expression in muscle is heterogeneous, with synaptic myonuclei expressing genes that regulate synaptic function and extrasynaptic myonuclei expressing genes to regulate contractile function, we used quantitative PCR to compare gene expression in these two domains of muscle from three different mouse models of SBMA: the “97Q” model that ubiquitously expresses mutant human androgen receptor (AR), the 113Q knock-in (KI) model that expresses humanized mouse AR with an expanded glutamine tract, and the “myogenic” model that overexpresses wild-type rat AR only in skeletal muscle. We were particularly interested in neurotrophic factors because of their role in maintaining neuromuscular function via effects on both muscle and synaptic function, and their implicated role in SBMA. We confirmed previous reports of the enriched expression of select genes (e.g., the acetylcholine receptor) in the synaptic region of muscle, and are the first to report the synaptic enrichment of others (e.g., glial cell line-derived neurotrophic factor). Interestingly, all three models displayed comparably dysregulated expression of most genes examined in both the synaptic and extrasynaptic domains of muscle, with only modest differences between regions and models. These findings of comprehensive gene dysregulation in muscle support the emerging view that skeletal muscle may be a prime therapeutic target for restoring function of both muscles and motoneurons in SBMA.

## 1. Introduction

Spinal bulbar muscular atrophy (SBMA) is a neuromuscular disease that causes muscle weakness and atrophy leading to a slow, progressive loss of motor function [1]. SBMA is linked to a polyglutamine expansion mutation in the *androgen receptor* (*AR*) gene [2]. There is currently no known cure. Men carrying the mutation develop the disease, while women carriers do not, although subclinical symptoms such as muscle cramping are common among female carriers. This sex difference in disease susceptibility is likely related to sex differences in circulating androgens, with male-typical levels driving the disease [3,4,5,6,7].

SBMA has classically been considered a “motoneuron” disease, but data from genetically engineered mouse models make it clear that AR acting solely in skeletal muscles can instigate significant, if not the full spectrum of neuromuscular pathology in SBMA [5,8,9]. Toxic AR in muscle induces three notable disease outcomes: (1) a profound loss of intrinsic muscle force that is independent of muscle mass [10,11], (2) impaired retrograde axonal transport in innervating motoneurons [12,13], and (3) defects in neuromuscular transmission involving impairments in neurotransmitter release [14,15]. Thus, it is critical to understand which genes in muscle underlie muscle dysfunction on the one hand, and motoneuron dysfunction on the other.

Published data indicate that gene expression in whole skeletal muscle from mouse models of SBMA is severely affected, showing large-scale transcriptome dysregulation [16,17,18]. However, it is not clear whether this dysregulation is relevant to all regions of the muscle. Skeletal muscle fibers are very large multinucleated cells in which gene expression differs across the length of the fiber. Nuclei situated at the synapse—the so-called “soleplate nuclei”, where a motoneuron innervates the fiber—express genes specialized for the synapse, while myonuclei outside the neuromuscular junction (NMJ) do not [19]. Because the mechanisms regulating gene expression differ in these two myodomains, a complete understanding of muscle dysfunction in SBMA requires gauging whether disease has a differential impact on synaptic versus extrasynaptic regions of muscle. This possibility seems plausible, because AR itself is preferentially expressed by synaptic myonuclei [20,21]. Here, we test this hypothesis by examining how disease affects gene expression in the synaptic versus extrasynaptic regions of muscle in three well-characterized mouse models of SBMA. These models include the “97Q” model, characterized by ubiquitous expression of a mutant human AR [3], the 113Q knock-in (KI) model expressing a humanized mouse AR with an expanded glutamine tract [6], and the “myogenic” model characterized by muscle-specific expression of wild-type (WT) rat AR [5]. SBMA mice in each model exhibit common disease traits that include an androgen-dependent loss of motor function in the absence of motoneuronal cell death that is associated with perturbed expression of neurotrophic factors in muscle, impaired motoneuronal retrograde axonal transport, and synaptic and muscle electrophysiological dysfunction [12,14,15].

Using quantitative real-time PCR, we examined the expression of a number of genes with known function. Our primary interest was in the neurotrophic factors, since such factors are implicated in SBMA and are critical for proper synaptic and muscle function, both of which are profoundly impaired in models of SBMA [5,6,10,11,14,15,22,23,24,25,26,27,28]. While skeletal muscles are well known to express numerous neurotrophic factors, including brain-derived neurotrophic factor (BDNF) and other related neurotrophins, it not known whether their expression is regionally regulated across the length of the fiber, particularly in the context of neuromuscular disease. Of the neurotrophic factors examined, we were surprised to find that most are comparably expressed in the two different regions of muscle, and that disease affects such genes in largely the same manner in both muscle domains. This finding raises the possibility that defects in the expression of neurotrophic factors critically mediate two core defects in SBMA—loss of muscle contractile strength and synaptic dysfunction. Thus, supplying neurotrophic factors broadly to muscles has the potential to restore function to both skeletal muscles and motoneurons affected by SBMA.

## 2. Results

**Figure 1:**
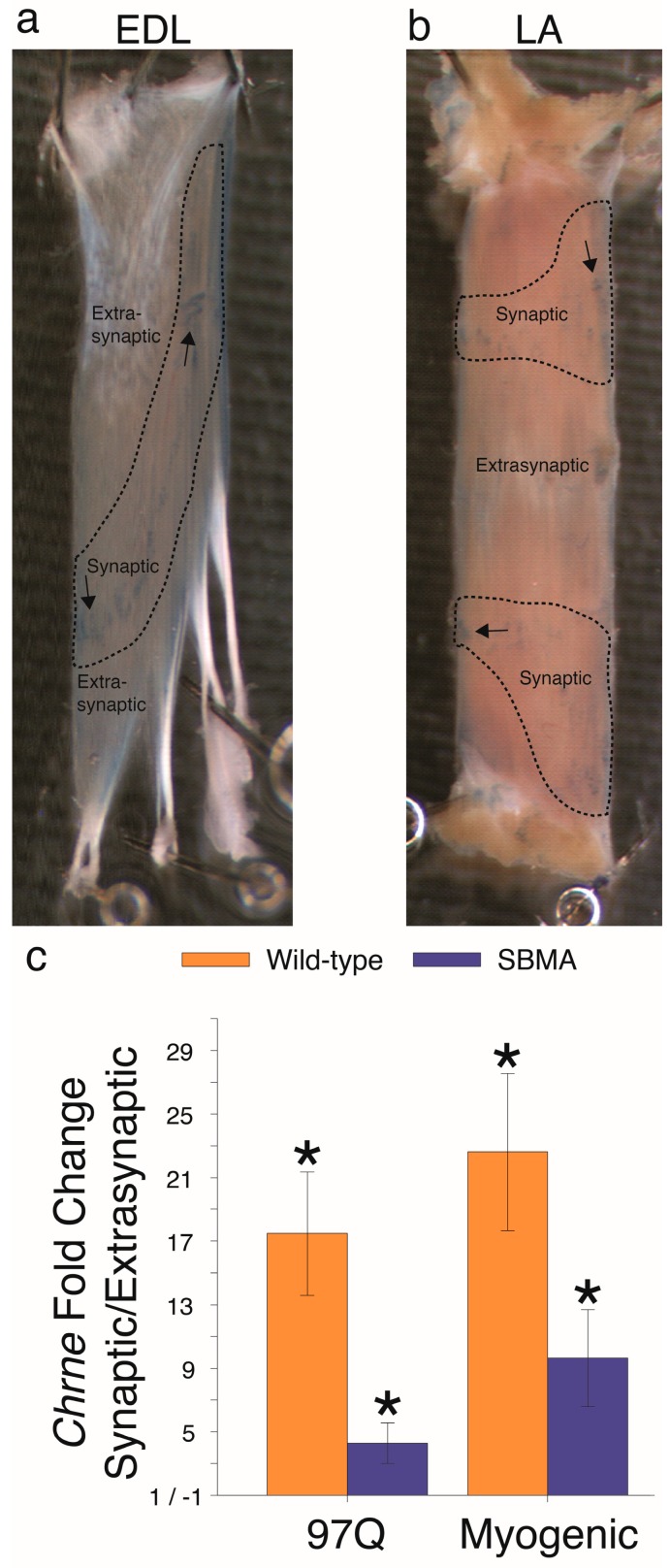
Levels of acetylcholine receptor epsilon subunit mRNA are significantly enriched in the synaptic region of WT and diseased muscle from two different SBMA mouse models. (**a**,**b**) The endplate, or synaptic region, of the muscle was localized by staining endplates (shown in blue, arrows) for cholinestrase (indicated by areas marked by dashed outlines). (**c**) The ratio of synaptic relative to extrasynaptic levels of *Chrne* transcripts in samples from WT and diseased muscle indicates that the synaptic region of the muscle contained as expected significantly higher levels of *Chrne* mRNA than the extrasynaptic region, confirming that our dissection method was valid and reliable for identifying synaptic- versus non-synaptic regions of muscle. Note however that this ratio was markedly smaller for diseased muscle than their respective WT controls. This reduced ratio reflects a net loss of *Chrne* transcript in the synaptic region of muscle rather than a significant increase in transcript levels in the extrasynaptic domain (see text), contrary to a denervation phenotype. These same samples were used to examine expression of other genes. Extensor digitorum longus, EDL; levator ani, LA. Fold changes ± SEM are relative to extrasynaptic samples. Statistical analysis was based on pair-wise fixed reallocation randomization test: * *p* < 0.05 for fold change in mRNA in synaptic relative to extrasynaptic.

The extensor digitorum longus (EDL) was examined in the myogenic and 97Q mice once they reached the same performance deficit (<30 s hang time), thus allowing us to relate changes in gene expression across different models to comparable levels of dysfunction rather than age. To verify that our dissection successfully separated synaptic and extrasynaptic regions, we measured mRNA levels for the adult epsilon subunit of the acetylcholine receptor (AChR) *Chrne*, which is normally enriched in the synaptic region of muscle [26]. We indeed found that synaptic samples contained a significantly higher level of *Chrne* transcript relative to extrasynaptic regions from the same set of muscles. Muscles from both healthy control and SBMA mice showed the same pattern of *Chrne* enrichment in the synaptic part of muscle (Figure 1). However, *Chrne* mRNA was appreciably lower in the synaptic region of diseased muscle relative to WT controls (97Q: −2.02 ± 0.34, *p* = 0.003; myogenic: −2.98 ± 0.64, *p* = 0.004). In contrast, *Chrne* transcript level was comparable in the extrasynaptic region of diseased and WT muscle (97Q: 2.02 ± 0.67, *p* = 0.064; myogenic: −1.27 ± 0.40, *p* = 0.463), consistent with earlier published work on these models indicating that diseased muscles are not denervated in end-stage mice [14,15,29], and that denervation per se does not cause a progressive loss of motor function in SBMA. Thus, the disease-related downregulation of this transcript, previously reported for whole muscle [14], reflects a loss specifically in the synaptic region, presumably contributing to the decline in synaptic strength seen in these models.

**Figure 2:**
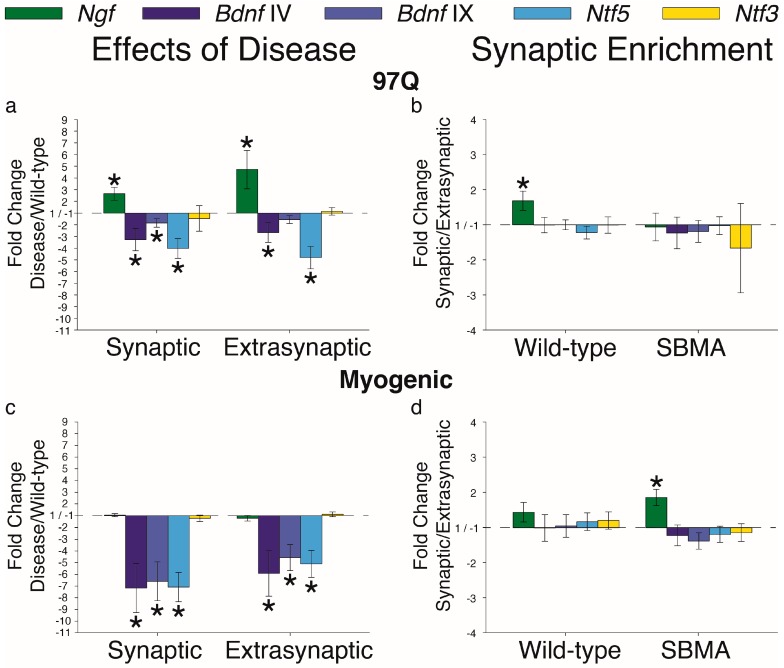
Neurotrophin mRNA expression in synaptic and extrasynaptic regions of skeletal muscle is largely comparably affected by disease. (**a**,**c**) *Ngf* was upregulated in the 97Q model but not in the myogenic model, possibly reflecting specific effects of a polyglutamine expanded AR expressed only in the 97Q model. On the other hand, *Bdnf* IV and IX variants were downregulated in both the 97Q and myogenic models, as we have previously reported. *Ntf5* was also downregulated in both models, aligning with previous findings in the knock-in model. Notably, *Ntf3* was not affected by disease in either model, indicating that disease affects most, but not all, of the neurotrophins expressed in skeletal muscle. (**b**,**d**) There were no consistent differences in neurotrophin mRNA expression between synaptic and extrasynaptic regions for either WT or diseased muscle from the two models examined. Fold changes ± SEM are relative to wild-type (**a**,**c**,**e**) or extrasynaptic (**b**,**d**,**f**) samples; Statistical analysis was based on pair-wise fixed reallocation randomization test: * *p* < 0.05.

Our next goal was to examine mRNA expression for the neurotrophins—nerve growth factor (NGF, *Ngf*), brain-derived neurotrophic factor (BDNF, *Bdnf*), neurotrophin-4/5 (NT-4, *Ntf5*), and neurotrophin-3 (NT-3, *Ntf3*). As found previously, whole muscle expression of both *Bdnf* [22] and *Ntf5* [6] were affected by disease and we now show that this indeed occurs in both domains (Figure 2a,c). Moreover, both neurotrophic factors were expressed uniformly across muscle domains in WT and diseased mice (Figure 2b,d). Two primer sets were used to examine *Bdnf* mRNA expression—one detecting only *Bdnf* transcript IV [30] and another recognizing a common region in exon IX, hence, detecting total *Bdnf* transcript. Levels of both IV and total *Bdnf* transcripts, as well as *Ntf5*, were downregulated in both synaptic and extrasynaptic regions of muscle from both 97Q and myogenic models. Of the remaining neurotrophins, disease had no effect on *Ntf3*, but triggered an upregulation of *Ngf* in muscle from 97Q mice, but not myogenic mice. Broadly speaking, there does not appear to be region-specific (synaptic or extrasynaptic) expression of any neurotrophin under healthy conditions (Figure 2b,d). That we found a statistically significant synaptic enrichment of *Ngf* may be a spurious observation, as it was seen in healthy WT mice of only one model, even though both are on a C57Bl/6J background.

**Figure 3:**
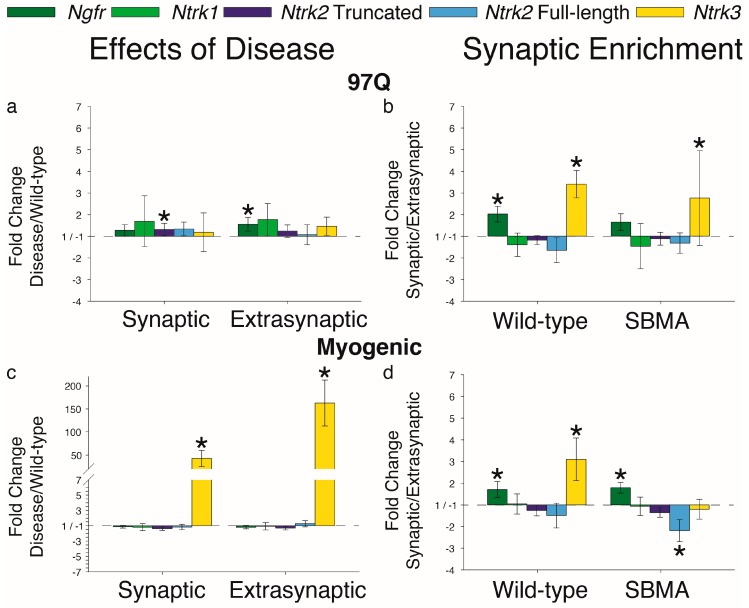
Disease has regionally specific effects on neurotrophin receptor expression. (**a**,**c**) *Ngfr*, truncated *Ntrk2,* and *Ntrk3* showed effects of disease, although the effects depended on the specific model. Disease upregulated truncated *Ntrk2* only in the synaptic region of muscle from 97Q but not myogenic mice. Disease robustly upregulated *Ntrk3* in both regions of muscle from myogenic but not 97Q mice. (**b**,**d**) In wild-type control muscle, *Ngfr* and *Ntrk3* were preferentially expressed in the synaptic region. Disease eliminated the synaptic enrichment of *Ngfr* in the 97Q model and of *Ntrk3* in the myogenic model. In sum, expression of neurotrophin receptors in muscle is also susceptible to the toxic effects of a disease-causing androgen receptor, and likely contribute to the pathophysiology of synaptic and muscle dysfunction in SBMA. Fold changes ± SEM are relative to wild-type (**a**,**c**) or extrasynaptic (**b**,**d**) samples; Statistical analysis was based on pair-wise fixed reallocation randomization test: * *p* < 0.05.

We next determined whether the receptors for the neurotrophins were affected by the disease, since that too could impair signaling. Previous data indicated that neither *Ngfr* nor *Ntrk2* mRNA expression was affected by disease in muscle of myogenic and 97Q mice [22]. However, when muscle region was considered in this study, a disease-related increase in truncated *Ntrk2* transcripts was detected only in the synaptic region of 97Q muscle (Figure 3a). Disease also triggered a significant upregulation of *Ngfr* in the extrasynaptic region of 97Q muscle. While neither *Ngfr* nor *Ntrk2* were affected by disease in the myogenic model, *Ntrk3* transcripts were robustly upregulated in both the synaptic and extrasynaptic regions of muscle in this model (Figure 3c). Disease did not affect full-length *Ntrk2* or *Ntrk1* in either region of muscle in either model.

We discovered that *Ntrk3* was enriched in the synaptic region of the EDL (Figure 3b,d). The disease eliminated this enrichment but only in the myogenic model. We also found an enrichment of *Ngfr* in the synaptic regions of the EDL muscle (Figure 3b,d), in line with a trend previously reported for the diaphragm [26]. Finally, the synaptic expression of full-length *Ntrk2* was reduced by disease in muscle from myogenic but not 97Q mice (Figure 3d).

**Figure 4:**
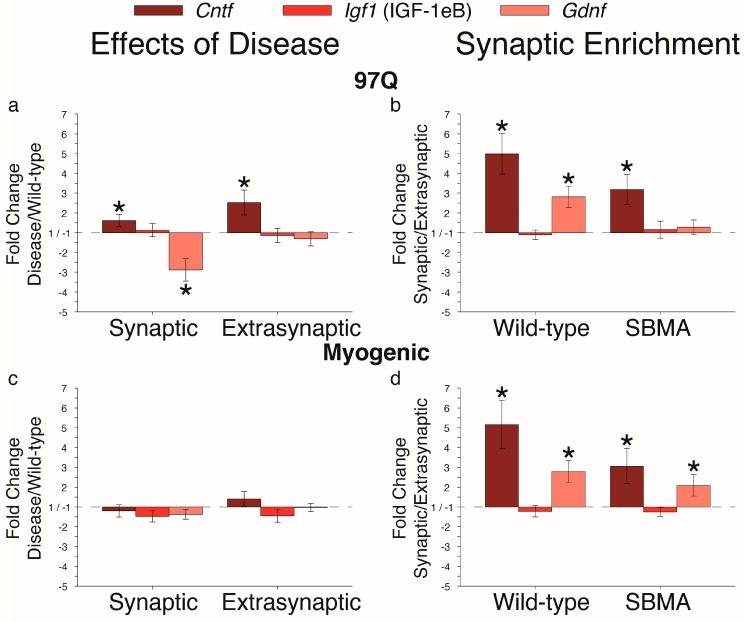
Disease affects expression of neurotrophic factors *Cntf* and *Gdnf,* but not *Igf1* (IGF-1eB variant). (**a**,**c**) Disease upregulated *Cntf* mRNA in muscle from 97Q but not myogenic mice. (**b**,**d**) Unlike the neurotrophins (Figure 2), expression of *Cntf* and *Gdnf* mRNA was enriched in the synaptic region of WT muscle, with disease having eliminated the synaptic enrichment of *Gdnf* in the 97Q model. Effects only in the 97Q model may reflect differences in the disease allele expressed (human disease allele AR in the 97Q model versus overexpression of WT rat AR in the myogenic model), and suggests that both *Cntf* and *Gdnf* may also contribute to neuromuscular dysfunction in SBMA patients. Fold changes ± SEM are relative to wild-type (**a**,**c**) or extrasynaptic (**b**,**d**) samples; Statistical analysis was based on pair-wise fixed reallocation randomization test: * *p* < 0.05.

To explore whether other neurotrophic factors are affected by disease, we chose to examine *Cntf*, which has been implicated in amyotrophic lateral sclerosis [31] and androgen-dependent motoneuron survival [32]. We also examined *Igf1* and *Gdnf* expression, each linked to SBMA [6,23,24]. Diseased 97Q, but not myogenic mice, showed an upregulation of *Cntf* transcripts in both regions of muscle (Figure 4a,c). *Igf1* transcripts (variant IGF-1eB) showed no effects of disease in either disease model. On the other hand, *Gdnf* mRNA was robustly downregulated by disease only in the synaptic region of muscle from 97Q, but not myogenic mice.

In comparing synaptic to extrasynaptic regions (Figure 4b,d), we found that *Cntf* mRNA levels were significantly, and consistently enriched in the synaptic region compared to the extrasynaptic region in both WT and diseased muscle of both models. This synaptic enrichment is likely due to *Cntf* mRNA expression by terminal Schwann cells [33]. Finally, *Gdnf* mRNA was also enriched in the synaptic region of both healthy and diseased muscle.

**Figure 5:**
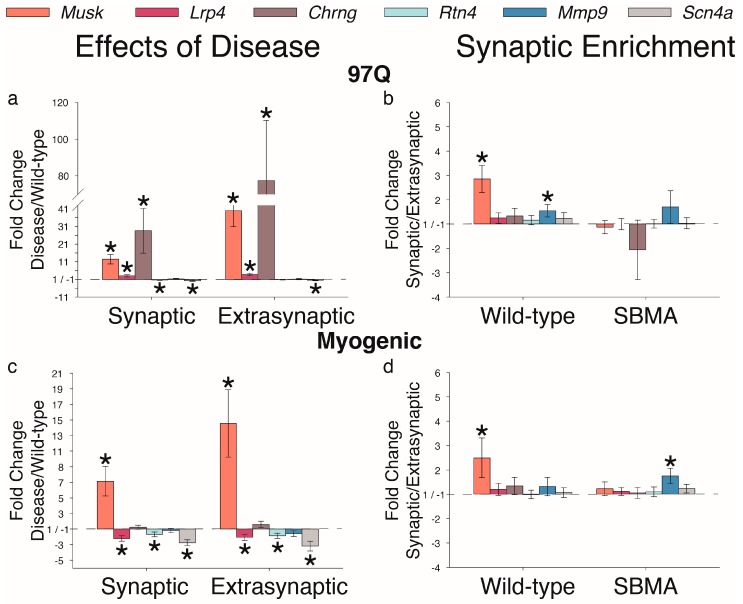
Disease affects genes implicated in synaptic stability and function. (**a**,**c**) With few exceptions, synaptic-related genes are affected by disease in one or both disease models. Changes include a marked increase in *Musk*, which encodes a receptor tyrosine kinase for the AChR-stabilizing agrin, and *Chrng*, encoding the neonatal subunit of the AChR. Disease also affects *Lrp4* in both models although in divergent directions—being increased in the 97Q model while decreased in myogenic model. Other significant and consistent changes in transcript levels include a downregulation of *Rtn4*, encoding an inhibitory signal for axonal sprouting, and *Scn4a*, encoding the sodium voltage-gated channel alpha subunit 4, the adult isoform which controls the influx of sodium into muscle cells. (**b**,**d**) Notably, the synaptic enrichment of *Musk* is also lost with disease. Thus, the challenges of disease seem to trigger an adaptive response to maintain and/or rescue neuromuscular synaptic function by reverting back to a permissive environment for axon sprouting (by downregulating *Rtn4)* and by increasing AChR expression and recruiting and stabilizing these newly made AChRs at the synapse (by upregulating *Musk* and *Chrng*). These data underscore the general theme that many genes important for synaptic stability are dysregulated in muscles of SBMA mice, possibly explaining the characteristic deficit in synaptic strength. Fold changes ± SEM are shown relative to wild-type (**a**,**c**) or extrasynaptic (**b**,**d**) samples; Statistical analysis was based on pair-wise fixed reallocation randomization test: * *p* < 0.05.

We also examined other genes implicated in synaptic structure and function, including *Musk*, *Lrp4*, *Chrng*, *Rtn4*, *Mmp9*, and *Scn4a* (Figure 5a,c,e). *Musk* mRNA was upregulated in both models, while the gene encoding its binding partner *Lrp4* was upregulated in the 97Q model, but downregulated in the myogenic model. We also found that *Rtn4* (encoding Nogo-A, an inhibitory signal for axonal sprouting) transcripts were reduced in myogenic and 97Q mice, hinting at the muscle’s attempt to re-establish strong synapses. Levels of *Chrng* mRNA, encoding AChR gamma subunit, were upregulated, but only in the 97Q model. *Mmp9* mRNA levels were not affected in these two models. *Scn4a* encoding the adult isoform of the alpha subunit of the voltage-gated sodium channel was significantly reduced in muscle from both 97Q and myogenic mice (Figure 5), confirming previous reports [14]. We found this reduction in both synaptic and extrasynaptic regions. Finally, disease eliminated a synaptic enrichment of *Musk* in muscle in both models, suggesting a possible mechanism leading to the dispersal of AChR (Figure 5b,d), which could account for previously reported fragmentation of endplates in diseased muscles [29].

We next examined expression of the same genes in a knock-in (KI) mouse model which expresses a polyglutamine expanded AR in its endogenous site driven by endogenous promoters [6]. The goal was to determine whether comparable gene dysregulation occurs in an SBMA model which has greater face validity than either transgenic model. We used the androgen-sensitive levator ani (LA) muscle (Figure 1b); ARs are highly expressed in this muscle relative to limb muscles such as the EDL [20,21], thus allowing the detection of pathology that might otherwise go undetected in this overall milder, earlier-staged model. Nonetheless, the LA is an excellent muscle to compare to the EDL, as the fiber-type between the two is virtually identical.

**Figure 6:**
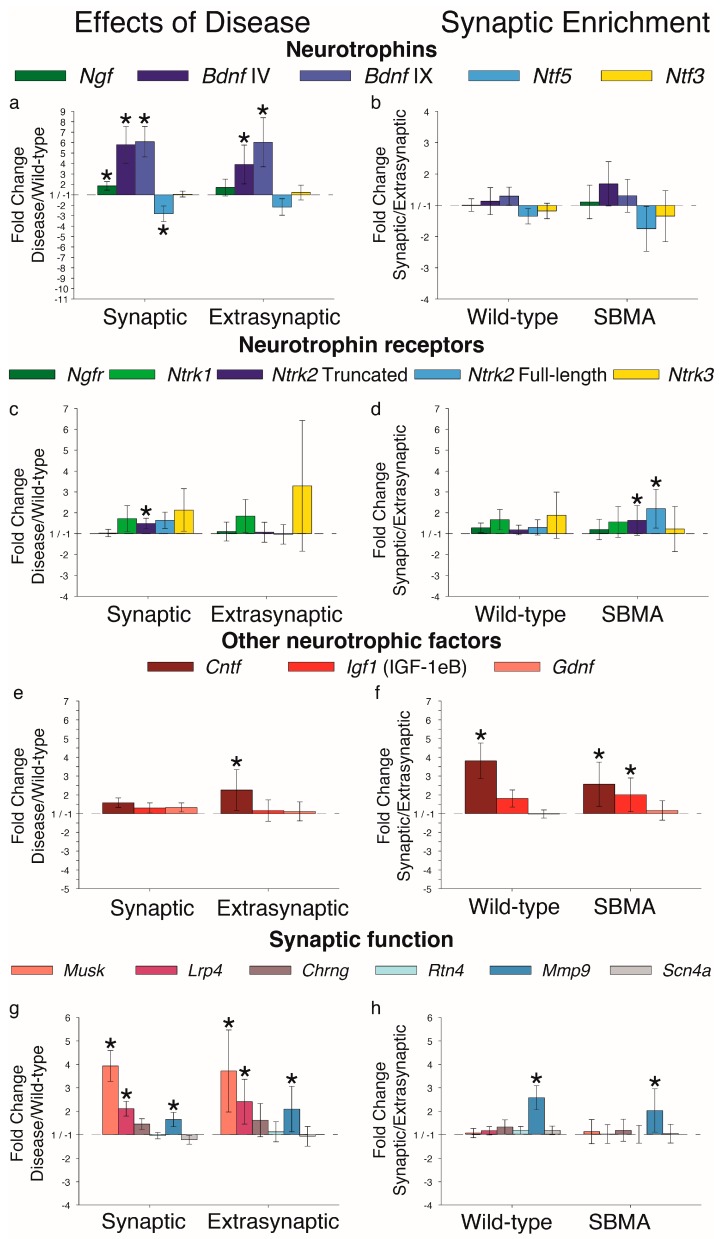
Disease affects many of the same genes in the fast twitch levator ani (LA) muscle from knock-in (KI) mice. (**a**) Neurotrophin (*Ngf*, *Bdnf*, and *Ntf5*) expression was disrupted by disease, largely comparable to what was seen for the fast twitch EDL of diseased 97Q and myogenic males. One notable difference was an upregulation of *Bdnf* induced by disease rather than the expected downregulation, as seen for the other two models. That *Bdnf* message is upregulated in muscle from diseased KI males may be a characteristic trait of early-stage disease. (**b**) Neurotrophin expression was not synaptically enhanced in muscle from KI males, largely mimicking what was seen in the other two models. (**c**) An upregulation of the truncated TrkB receptor transcript was also observed only in the synaptic region, paralleling results for the 97Q model. (**d**) Interestingly, the wild-type LA did not show a synaptic enrichment of either *Ngfr* or *Ntrk3* as was observed for the EDL of the other two models. Disease disrupted this uniform expression in the LA, such that *Ntrk2* mRNA became synaptically enriched. (**e**) Of the other neurotrophic factors examined, *Cntf* was the only one dysregulated by disease, a novel finding implicating Schwann cells in SBMA. A downregulation of *Igf1* and *Gdnf* was not detected as previously reported for KI mice, possibly due to the fewer number of CAGs carried by this later generation of KI mice. (**f**) *Cntf* was synaptically enriched in the LA, as in the EDL. (**g**) *Musk* and *Lrp4* transcripts were also dysregulated, comparable to results from the myogenic and 97Q models. However, these changes involved both regions, an unexpected finding given the established role of *Musk* and *Lrp4* in synaptic function. *Mmp9* was also affected in muscle from KI mice. (**h**) That it was also synaptically enriched in both WT and diseased LA may reflect a specific trait of this muscle, since this synaptic enrichment was not evident in the EDL. Fold changes ± SEM are shown relative to wild-type (**a**,**c**,**e**,**g**) or extrasynaptic (**b**,**d**,**f**,**h**) samples; Statistical analysis was based on pair-wise fixed reallocation randomization test: * *p* < 0.05.

We again verified the synaptic enrichment of *Chrne*, with WT mice showing 10.1 ± 2.8 fold upregulation (*p* = 0.001) and diseased mice showing 7.8 ± 4.6 upregulation (*p* = 0.001) compared to the extrasynaptic region. In our assessment of the expression of neurotrophins and their receptors, we found that, like the 97Q model, *Ngf* was upregulated by disease in KI mice, but only in the synaptic region (Figure 6a). That this was not found in the myogenic mice possibly reflects the fact that the 97Q and KI models both express an expanded polyglutamine tract in AR that the myogenic model does not. *Bdnf* was also affected by disease in the KI model, but surprisingly it was *upregulated* (rather than downregulated) in KI muscle. Since the KI model exhibits relatively less severe pathology, this upregulation may reflect an earlier stage in the disease process. On the other hand, *Ntf5* mRNA levels were significantly downregulated by disease, as in the 97Q and myogenic mice, but only in the extrasynaptic region of KI muscle. Finally, *Ntf3* expression was not affected, mimicking results in muscle from 97Q and myogenic mice. For neurotrophin receptors, muscle from KI mice showed upregulation of truncated *Ntrk2* only in the synaptic region (Figure 6c), as was the case for the 97Q model. The full-length *Ntrk2* was not perturbed by disease when compared to WT muscle, but KI mice did exhibit a synaptic enrichment that was not present in WT mice suggesting that disease did affect this variant as well (Figure 6d).

Other neurotrophic factors were also affected in the KI model, in alignment with our findings in the other two models. *Cntf* was upregulated in KI muscle as it was in 97Q muscle, but the effect was significant only in the extrasynaptic region (Figure 6e). That this effect was not found in the myogenic model suggests it is mediated by mutant, polyglutamine-expanded AR which is present in the KI and 97Q models. *Cntf* was also enriched synaptically (Figure 6f), as found previously (Figure 4). Neither *Igf1* nor *Gdnf* were affected by disease in the KI model. The null *Gdnf* finding may reflect a loss of CAG repeats over generations, or muscle-specific changes as mice from earlier in the lineage did express lower *Gdnf* transcript levels than controls in hindlimb muscle [6].

Muscle from KI mice also showed an enhanced expression of *Igf1* in the synaptic region of the muscle, while muscle from the other models did not, possibly reflecting a specific feature of the LA not shared by the EDL. While this difference was only significant for diseased muscle, the magnitude of the effect was similar in WT muscle, suggesting that the failure to detect a significant difference in WT muscle (*p* = 0.076) reflects a type II error.

Muscle from the KI model also exhibited some dysregulation in the expression of synaptic-related genes (Figure 6g). For example, like the other two models, *Musk* levels were upregulated. *Lrp4* was also upregulated, mimicking the pattern seen in 97Q mice. Interestingly, *Mmp9* levels were increased by disease uniquely in the KI model in both the synaptic and extrasynaptic regions. In sum, this humanized mouse model of SBMA also indicates that disease disrupts muscle expression of genes that play key roles in muscle and synaptic function.

## 3. Discussion

We evaluated for the first time whether disease triggered by toxic AR affects gene expression differently in the synaptic versus the extrasynaptic regions of skeletal muscle. Intrigued by recent findings that AR in muscle has the capacity to drive significant dysfunction in both muscle and motoneurons in mouse models of SBMA [10,11,12,13,14], we asked whether gene expression in diseased muscle was impaired in a region-specific manner. This question was also prompted by the fact that myonuclei across the length of the muscle express different genes; specifically, myonuclei at the NMJ express synapse-specific genes that other myonuclei do not [19], demonstrating that different mechanisms control gene expression in these two parts of muscle. We addressed this question by examining gene expression in synaptic and extrasynaptic muscle from three different SBMA mouse models: the 97Q, the myogenic, and a KI model, with the goal of identifying core attributes of disease that could translate to patients with SBMA. Our analysis focused on neurotrophic factors for two reasons: (1) they have a well-established role in modulating both pre-synaptic motoneuronal function and post-synaptic muscle function [34,35,36], and (2) they are implicated in the etiology of neuromuscular disease, including SBMA [6,22]. Our findings aligned with other reports that disease does indeed affect the expression of multiple neurotrophic factors in skeletal muscle, including the neurotrophins and their receptors, as well as CNTF and GDNF, with disease generally affecting the expression of such genes comparably across the two muscle regions (Table 1). These findings revive the idea that neurotrophic factors may have therapeutic value for restoring function that could potentially benefit both diseased muscles and the motoneurons that innervate them. We also found that disease affected other key genes controlling synaptic stability and function, including effects on *Musk* and *Lrp4*, both of which help stabilize AChR in the muscle membrane. Other genes were discovered to show enhanced expression in the synaptic region of skeletal muscle, in addition to replicating earlier findings of genes already known to show synapse-enriched expression in muscle [26,37,38,39]. In most cases, this synaptic enrichment was preserved in the face of disease. Our cross-model comparisons converged on several genes affected by disease, most notably, the neurotrophins BDNF and NT-4. Because they showed comparable and robust dysregulation across models, they may represent bona fide pathophysiological mechanisms underlying SBMA in patients.

We found that BDNF/NT-4—TrkB signaling, a pathway that is important in neuromuscular transmission and muscle function [27,28,40], was quite susceptible to the effects of disease caused by a toxic AR. As previously reported, we found that *Bdnf* transcripts were robustly downregulated in muscle from both myogenic and 97Q mice. We now show that these changes do *not* depend on region of muscle; *Bdnf* was similarly affected by disease in both synaptic and extrasynaptic domains. We also found that *Bdnf* expression was affected in muscle of KI mice. However, in this model, the level of *Bdnf* message increased. As BDNF expression in muscle can have a time-dependent response to other trauma such as denervation, involving first an upregulation followed by a downregulation of expression [41,42,43,44], it is possible that the bidirectional effect of disease across models relates to the stage of disease, with KI muscle representing an earlier stage of disease than muscle from diseased 97Q and myogenic males. Supporting this idea is the fact that KI males tend to die early due to urinary tract blockage and uremia [6], with defects in neurotransmission reflecting primarily the early stages of disease [15]. End-stage myogenic or 97Q males, on the other hand, tend to exhibit more severe neuromuscular dysfunction involving more mechanisms, presumably reflecting the combined effect of both primary and secondary mechanisms of disease [15]. In sum, *Bdnf* expression in muscle was perturbed in all three models suggesting that its role in SBMA merits further attention.

Another TrkB ligand, encoded by *Ntf5* (NT-4), was downregulated in all models while transcript level for the truncated, but not the full-length TrkB receptor (encoded by *Ntrk2*) was upregulated in the 97Q and KI models and only in the synaptic region. One interpretation of these data is that elevated levels of truncated TrkB is a response to an ever-diminishing supply of muscle-derived BDNF and/or NT-4, attempting to maintain a constant supply of such factors to the motor terminals. However, the cost may be to further reduce both synaptic and muscle function, since both are enhanced in TrkB.T1 knockout mice [45]. Moreover, overexpression of TrkB.T1 results in NMJ fragmentation [46], comparable to what junctions look like in SBMA models [29].

Our findings of increased levels of *Musk* and *Chrng* transcripts replicate previously published data on whole muscle [14,47]. *Musk* encodes a tyrosine kinase receptor for agrin, an AChR clustering factor, whereas *Chrng* encodes the neonatal gamma subunit that forms the channel in the AChR. To our surprise, expression of both mRNA species was comparably dysregulated in synaptic and extrasynaptic regions of muscle, despite their apparent specific roles in synaptic function. Levels of *Scn4a*, encoding the sodium voltage-gated channel alpha subunit 4, were also perturbed in the 97Q and myogenic models, showing a net loss in *Scn4a* as previously shown [14]. We did not, however, find the expected deficit in *Scn4a* in muscle of KI males in this study, perhaps because of the shorter CAG repeat of the KI mice used in this study compared to the original [6], causing lower overall AR toxicity.

Of the remaining genes examined, we saw changes in all three models for only *Lrp4*, which, like *Musk*, encodes a protein that promotes AChR clustering. The dysregulation of these genes may explain the fragmented morphology of diseased NMJs in SBMA models [29]. MuSK is activated by Lrp4 following the binding of agrin to Lrp4 [48,49]. It is possible that there is an ongoing interplay with MuSK/Lrp4 and the TrkB signaling pathways due to their similar roles in synaptic stabilization. At the molecular level, both MuSK and Trk receptors are receptor tyrosine kinases. Indeed, replacement of MuSK with a chimeric MuSK/Trk (using the intracellular kinase domain of TrkA) is capable of restoring function and rescuing MuSK^−/−^ pups from perinatal lethality [50]. In myotube cultures, however, BDNF and NT-4 inhibited agrin-induced AChR clustering [51]. It is worth noting that *Lrp4* was downregulated in the myogenic model but upregulated in the others. Although it is difficult to understand the significance of these divergent responses in *Lrp4* across the three models, these data underscore the general theme that many genes important for synaptic stability are dysregulated in muscles of SBMA mice (Figure 5 and Figure 6), which could underlie the deficits in synaptic strength [14], a core attribute of disease in all three models.

The loss of the neurotrophin NGF has been linked to muscular dystrophy [52,53]. For example, treatment with NGF improves regeneration of dystrophic muscle cells [54]. Moreover, after denervation or tetrodotoxin-induced nerve blockage, *Ngf* transcripts increase in rat muscle [55]. That we also saw an upregulation of *Ngf* mRNA levels in two SBMA models (Figure 2 and Figure 6) suggests that disease causes NMJs to fall below threshold, effectively becoming functionally denervated [14], even in the absence of overt structural denervation [29]. That we did not see changes in *Ngf* expression in the myogenic model suggests that this disease-related change may stem from toxic AR acting neuronally.

Terminal Schwann cells (TSCs) are critical for proper neuromuscular transmission [56]. As TSCs express *Ntrk3* and *Cntf* [33,38], we attribute the synaptic enrichment of these genes to TSCs. An interesting pattern unfolded when examining effects of disease on expression of *Ntrk3* and *Cntf*. Myogenic mice showed a robust disease-related upregulation of *Ntrk3* in both synaptic and extrasynaptic regions of muscle, while the 97Q and KI mice showed upregulation of *Cntf* by disease. Thus, it is possible that TSCs are involved in SBMA pathophysiology, but perhaps through different pathways depending on the instigating genetic/molecular trigger. One potentially critical distinction between these models is that both the KI and 97Q models express an expanded AR while the myogenic model does not. It would be worthwhile to examine further the potential role of TSCs in SBMA, especially given the link between TSCs and other neuromuscular diseases, such as amyotrophic lateral sclerosis [57]. While TSC morphology largely resists the effects of disease, their function may be profoundly affected [29].

A limitation inherent to the approach in this study is that our samples did not contain solely muscle cells. Importantly, the synaptic samples undoubtedly contained TSCs, which could explain the enrichment of *Cntf* in this region. This nonetheless endows us with confidence that our harvest of the synaptic region was precise. In fact, by isolating and examining only muscle fibers, we might actually miss important changes that occur in other cell types within the muscle that contribute to the neuromuscular dysfunction associated with SBMA. Thus, when interpreting our results, which largely *do not* show distinct effects of disease across regions, we take it to mean that SBMA muscles are broadly affected. This pattern suggests that AR acts throughout the muscle to impair muscle function directly, and presynaptic motoneuronal function indirectly [5,6,9], and that many of the mechanisms involved may well be the same.

Our studies offer the power of a systematic, cross-model approach to understanding gene dysregulation in SBMA muscle. Studies that focus on a single model may be a factor contributing to the poor success rate of clinical trials [58]. Commonalities seen across three quite different mouse models of SBMA lend confidence to the idea that the identified targets are likely relevant to SBMA patients. Moreover, by examining genes regulated in a similar manner between severely affected 97Q and myogenic models, but not in the less affected KI model, we may gain insight into the molecular and cellular sequelae underlying aspects of disease progression and disease specificity caused by enhanced levels of AR protein, whereas similarities between the 97Q and KI models not shared by the myogenic model may shed light on the toxicity caused by the expanded polyglutamine tract. Regardless of the exact patterns of dysregulation, and their potential pathophysiological significance, we have shown that the majority of genes in the neurotrophin family (including both ligands and receptors) are affected by disease in SBMA skeletal muscle. An important next step will be to characterize how such changes at the mRNA level affect function, both regarding the specific protein products and how they interact to influence neuromuscular function. Neurotrophic factors, and possibly neurotrophins specifically, may be at the center of such disease-related changes.

## 4. Materials and Methods

### 4.1. Animals

Mouse colonies were held on a 12 h:12 h light:dark cycle, group housed, and provided food and water ad libitum. All animal procedures were approved and performed in compliance with Michigan State University’s (approval# 11/16-198-00, 30 November 2016) and University of Michigan’s (approval# PRO00008133, 24 January 2018) Institutional Animal Care and Use Committees in accordance with the standards in the NIH Guide for the Care and Use of Laboratory Animals. Sample sizes are listed in Table 2.

### 4.2. AR97Q Model

Transgenic male mice ubiquitously overexpressing a full length human AR with a 97 glutamine repeat and WT age-matched controls (age, mean ± SEM (range): 111.7 ± 10.1 (63–149) days) from the same colony were maintained on a C57Bl/6J genetic background. Mice were genotyped using PCR at weaning as previously described [3]. Muscle was harvested once the mice became symptomatic, showing reduced ability to perform on motor assays, as previously described [22].

### 4.3. Myogenic Model

Transgenic male mice overexpressing rat WT AR exclusively in skeletal muscle fibers and WT age-matched controls (age, mean ± SEM (range): 112.1 ± 3.9 (80–125) days) from the same colony were maintained on a C57Bl/6J genetic background. Mice were genotyped using PCR at weaning as previously described [5]. Transgenic and WT males were exposed prenatally to the anti-androgen flutamide (5 mg flutamide/0.1 mL propylene glycol at the nape of the neck on gestational days 15–20 [59]), to block the apparently toxic effects of prenatal endogenous androgens on postnatal survival. Muscle was harvested from symptomatic adults, which show reduced ability to perform on motor assays, as previously described [12,22].

### 4.4. AR113Q Knock-In (KI) Model

KI male mice expressing an expanded CAG allele (92–96 CAG repeat, determined for 4 of the 7 KI mice used in this study) in the first exon of the human *AR* gene and age-matched WT controls (age, mean ± SEM (range): 117.4 ± 4.5 (112–137, except one 65-day old WT)) from the same colony were maintained on a C57Bl/6J background [6].

### 4.5. Muscle Dissection

Skeletal muscle was dissected from mice anesthetized with isoflurane and immediately placed into ice-cold PBS. Muscles were rapidly pinned in a Sylgard-coated dish. The muscle was then divided into synaptic and extrasynaptic regions based on considerable prior experience recording synaptic transmission in these muscles [14,15], and collected in pre-chilled microcentrifuge tubes and stored at −80 °C. Our dissection was also based on experience with recording synaptic transmission in these muscles [14]. Care was taken to avoid collecting connective tissue and tendons. All tools were cleaned with RNase Zap (Ambion, Thermo Fisher Scientific Waltham, MA, USA) between animal harvests. The extensor digitorum longus (EDL) was harvested for the myogenic and 97Q models, while the androgen-sensitive levator ani (LA) was collected for the AR113Q KI model. The effects of disease are more readily detected in the LA than in other skeletal muscles [6], likely due to the much higher level of AR expression in this muscle [20,21]. Both the EDL and LA muscles are fast twitch muscles [60,61,62,63,64], controlling for the possible differential effects of disease introduced by different fiber-types.

### 4.6. Quantitative Reverse-Transcription PCR

Muscles were mechanically homogenized with a PRO200 Homogenizer (Pro Scientific) in TRIzol reagent (Ambion). RNA was extracted according to manufacturer directions, with samples treated with DNase I (Invitrogen, Thermo Fisher Scientific Waltham, MA, USA). Following extraction, RNA was quantified on a spectrophotometer (DU 530, Beckman Coulter, Brea, CA, USA) by measuring 260 nm absorbance values and reverse transcribed using the High Capacity cDNA Reverse Transcription Kit (Applied Biosystems, Carlsbad, CA, USA) with the following thermocycle settings: 25 °C for 10 min, 37 °C for 2 h, 85 °C for 5 min. Each sample for the quantitative real-time PCR assay included 2.5 ng of cDNA, primers, and Power SYBR Green PCR Master Mix (Applied Biosystems). Thermocycle for the quantitative step on the ABI PRISM 7000 Sequence Detection System was as follows: 50 °C for 2 min, 95 °C for 10 min, and 40 cycles of 95 °C for 15 s and 60 °C for 1 min. A dissociation curve was determined for each well to confirm that only one gene was being amplified. Each sample was run in triplicate. Samples without reverse transcriptase during the cDNA conversion were also assessed to ensure that there was no DNA contamination. Optimal concentrations and amplification efficiencies were calculated for each primer set. Primers, concentrations, and efficiencies are listed in Table 3. *Rn18s* was used as the control and diluted 100-fold due to its higher expression. Finally, we verified that *Rn18s* expression did not differ between any comparisons made.

### 4.7. Statistical Analysis

The Relative Expression Software Tool (REST) was used to assess statistical significance and fold change of genes [65]. Specifically, this software uses the non-parametric Pair-Wise Fixed Reallocation Randomisation Test to account for amplification efficiencies when determining fold change. It measures relative expression of a target gene between two experimental groups following the normalization of the target gene to a reference gene (*Rn18s*).

Our experimental groups consisted of the SBMA mouse models and their WT controls taken from each respective colony. For each model, we asked the same two questions: (1) are there regional differences in gene expression (synaptic versus extrasynaptic), and (2) does disease affect gene expression comparably in the two regions?

## Figures and Tables

**Table 1:** Cross model comparison of disease-affected genes from this study. * indicates differential effects of disease on synaptic and extrasynaptic regions. ^#^ indicates directional difference from other models.

	*Chrne*	*Ngf*	*Bdnf* (IV)	*Bdnf* (IX)	*Ntf5*	*Ntf3*	*Ngfr*	*Ntrk1*	*Ntrk2* (Truncated)	*Ntrk2* (Full)	*Ntrk3*	*Cntf*	*Igf1*	*Gdnf*	*Musk*	*Lrp4*	*Chrng*	*Rtna*	*Mmp9*	*Scn4a*
97Q	✔	✔	✔	✔	✔		✔ *		✔			✔		✔ *	✔	✔	✔	✔		✔
Myogenic	✔		✔	✔	✔						✔				✔	✔ ^#^		✔		✔
KI		✔	✔ ^#^	✔ ^#^	✔				✔			✔			✔	✔			✔	

**Table 2:** Sample sizes (n) by model and muscle region, *n* = number of animals.

	Wild-Type	SBMA
**97Q**		
Synaptic	5	6 (5 for *Gdnf*; 4 for *Rtn4* and *Ntrk1*)
Extrasynaptic	5	6 (4 for *Gdnf, Rtn4,* and *Ntrk1*)
**Myogenic**		
Synaptic	6	7
Extrasynaptic	7	8
**Knock-in**		
Synaptic	7	6
Extrasynaptic	7	6 (5 for *Ntrk1*)

**Table 3:** Primer sequences and concentrations used for genes examined.

Gene	Forward	Reverse	Calculated Efficiency	Concentration (nM)
*Rn18s* (18S)	GGACCAGAGCGAAAGCATTTG	GCCAGTCGGCATCGTTTATG	1.90	100
*Chrne* (AChR epsilon)	CTCTGCCAGAACCTGGGTG	TGTGCTCTCAGCCACAAAGT	2.15	200
*Ngf*	AGCTTTCTATACTGGCCGCA	TACGCCGATCAAAAACGCAG	1.92	600
*Bdnf* (exon IV)	CTCCGCCATGCAATTTCCAC	CGAGTCTTTGGTGGCCGATA	1.74	200
*Bdnf* (exon IX)	ACCATCCTTTTCCTTACTATGGTT	ATTCACGCTCTCCAGAGTCC	1.98	200
*Ntf5* (NT-4)	TGAGCTGGCAGTATGCGAC	CAGCGCGTCTCGAAGAAGT	2.03	600
*Ntf3* (NT-3)	TGGAGCCCCCTCCCTTATAC	AATGGCTGAGGACTTGTCGG	2.23	100
*Ngfr* (p75)	CGTGACCATCTCAGGCCTTT	GGTGCCCCTGTTACCTTCTC	2.01	200
*Ntrk1* (TrkA)	ATATCTAGCCAGCCTGCACTTTGT	TGCTCATGCCAAAGTCTCCA	2.15	600
*Ntrk2* (TrkB truncated)	CCATTGCCCTCTGCTAACCA	GAGATCTGAGGTGCTCTCGC	2.08	600
*Ntrk2* (TrkB full length)	GGCAACTTCGGGAAAGGAGA	GTAAACCCCTCACCGCCTAC	2.25	400
*Ntrk3* (TrkC)	ATGGAGCTCTACACGGGACT	GGTGAGCCGGTTACTTGACA	2.40	600
*Cntf*	TTTCACCCCGACTGAAGGTG	TTCTGTTCCAGAAGCGCCAT	2.10	200
*Igf1* (IGF-1eb)	CCCGTCCCTATCGACAAACAA	TGGGAGGCTCCTCCTACATT	2.00	100
*Gdnf*	GCCACCATTAAAAGACTGAAAAGG	GCCTGCCGATTCCTCTCTCT	1.91	600
*Musk*	GCTGTTTGACACCCGCTACA	CTCCCACTCCATTGTTGGCTA	1.97	400
*Lrp4*	GCATTGGTACTGCGATGGTG	CATAGGCGCACTGGAACTCT	1.94	100
*Chrng* (AChR gamma)	GGTTGGTGATGGGTATGGTCA	TGACATCAGGAAAGGCAGAGC	2.06	200
*Rtn4* (Nogo-A)	ACTTACGTTGGTGCCTTGTTC	TGATCTATCTGCGCCTGATGC	1.67	200
*Mmp9*	GCCGACTTTTGTGGTCTTCC	CTTCTCTCCCATCATCTGGGC	2.01	200
*Scn4a* (NaV1.4)	TGGGGGTCAACTTGTTTGCT	TCGAATCTCTCGGAGGTGGT	2.09	100
